# Supplementary material for: Determinants of community members’ willingness to donate stool for faecal microbiota transplantation
Source: PLoS One. 2020 Dec 10;15(12):e0243751. doi: 10.1371/journal.pone.0243751 (PMC7728237; doi:10.1371/journal.pone.0243751)
Supplement: S1 Appendix — (DOCX) [file pone.0243751.s001.docx]

**S1 Appendix. Pre-screening and Main Surveys**

**PRE-SCREENING SURVEY**

**Participant Characteristics**

1. What is your age (in years, e.g., 23)?

________________________________________________________________

1. Do you consider yourself to be in good health with no current medical conditions?

⭘ Yes

⭘ No

⭘ Unsure

1. Do you consider yourself to be of normal weight?

⭘ Yes

⭘ No

⭘ Unsure

1. Are you currently taking medication for a medical condition?

⭘ Yes

⭘ No

⭘ Unsure

1. To the best of your knowledge, are you eligible to donate blood? You can check your eligibility here {opens in a new tab <https://www.donateblood.com.au/eligibility>}

⭘ Yes

⭘ No

⭘ Unsure

1. Do you have a condition or illness that affects your bowel movements?

⭘ Yes

⭘ No

⭘ Unsure

**If ineligible:**

Thank you for participating in this short task. Based on your responses you are not eligible for our main study. Please proceed to the next question and your account will be credited for your participation in this task.

**If eligible:**

Thank you for participating in this short task. Based on your responses, you will soon be invited to participate in our main study: **Understanding Community Attitudes Towards Stool Donation**. Please proceed to the next question and your account will be credited for your participation in this task.

**MAIN SURVEY**

**Participant Characteristics**

1. What is your age (in years, e.g., 23)?

________________________________________________________________

1. With which gender do you identify?

⭘ Male

⭘ Female

⭘ Other, please specify how you identify below

________________________________________________________________

1. What is the highest level of education you have completed?

⭘ University

⭘ TAFE

⭘ High School

⭘ Primary School

⭘ Less than Primary School

⭘ Other, please specify below

________________________________________________________________

1. What is your height (rounded to the nearest cm)?

________________________________________________________________

1. What is your weight (rounded to the nearest kg)?

________________________________________________________________

1. Have you donated blood in the past year?

⭘ Yes

⭘ No

1. Have you registered to be an organ donor on the Australian Organ Donor Register (the national organ donor register)?

⭘ Yes

⭘ No – I haven’t got around to it

⭘ No – I do not want to donate my organs

⭘ Unsure

**Bowel Habits**

There is no recent research available on healthy Australian’s bowel habits and we would be very grateful if you could please answer the questions below as accurately as you can.

1. Do you have regular bowel movements?

⭘ Yes

⭘ No

⭘ Sometimes

1. Do you usually have a bowel movement every day?

⭘ Yes

⭘ No

⭘ Sometimes

1. On average, how many bowel movements do you have in a **day** (that is, how many times a day do you have a bowel movement)?

________________________________________________________________

1. On average, how many bowel movements do you have in a **week** (that is, how many times a week do you have a bowel movement)?

________________________________________________________________

1. Is the consistency of your stool (i.e., poo) smooth and shaped like a sausage?

⭘ Yes

⭘ No

⭘ Sometimes

⭘ Unsure

1. When in the day do you usually or most often have a bowel movement?

⭘ In the morning

⭘ Midday

⭘ In the afternoon

⭘ In the evening

⭘ At night

1. If you usually have **more than one** bowel movement in a day, when in the day do you usually or most often have another bowel movement?

⭘ In the morning

⭘ Midday

⭘ In the afternoon

⭘ In the evening

⭘ At night

⭘ Not applicable

1. On average, approximately how long from start to finish does it usually take you to do a bowel movement (in minutes)?

________________________________________________________________

**Bowel Movements in Public Restrooms**

1. These questions ask you about common (yet uncomfortable) experiences that people sometimes have when using the toilet in a public restroom. Please answer honestly.

|  | None of the time | A little of the time | Some of the time | Most of the time | All of the time |
| --- | --- | --- | --- | --- | --- |
| I cannot use the toilet in a public restroom to have a bowel motion when other people are around | ⭘ | ⭘ | ⭘ | ⭘ | ⭘ |
| I avoid going to the toilet in a public restroom, even if I need to have a bowel motion | ⭘ | ⭘ | ⭘ | ⭘ | ⭘ |
| I delay going to the toilet in a public restroom, even if I need to have a bowel motion | ⭘ | ⭘ | ⭘ | ⭘ | ⭘ |
| I worry I cannot empty my bowel in a toilet in a public restroom when others are nearby | ⭘ | ⭘ | ⭘ | ⭘ | ⭘ |

**Knowledge Part I**

1. Have you ever heard of an illness called Clostridioides difficile or C. difficile (***prior to this study***)?

⭘ Yes

⭘ No

⭘ Unsure

**Clostridioides difficile** (also referred to as Clostridium difficile, **C. difficile**, or *C. diff*) is a bacteria (germ) that normally lives in your intestines (gut). Sometimes, *C. difficile* grows out of control and can cause infection. Infection can result in frequent diarrhoea for more than a few days, feeling sick to your stomach, stomach pain, and fever. For most people the effects of a *C. difficile* infection are relatively mild, but it can sometimes lead to serious illness and even death. A *C. difficile* infection is normally treated with antibiotics. However, for some people, antibiotics don’t work or the infection returns many times. People who are most at risk for *C. difficile* infection include people who have taken antibiotics, are being treated for cancer, are staying in a hospital, live in a nursing home, have had surgery on their stomach or intestines, or have a weakened immune system.

1. Have you ever heard of faecal microbiota transplantation (also called a stool transplant or a poo transplant) (***prior to this study***)?

⭘ Yes

⭘ No

⭘ Unsure

**Faecal microbiota transplantation** (**FMT**) involves the transfer of stool from a healthy person to a person with disease in order to treat their disease. It can be a life-saving treatment for people who have recurring *C. difficile* infection. *FMT* has been shown in randomised controlled trials to cure up to 90% of people who have recurring *C. difficile* and for whom antibiotics do not work as a treatment. New research shows that *FMT* may also help to treat people with other conditions such as ulcerative colitis. During the transplant, pre-prepared stool from a carefully screened, healthy person who has donated stool to a stool bank, is transplanted into the colon of the patient. The transplant usually happens by colonoscopy or capsules. *FMT* is thought to work by replacing the patient’s gut microbiome (community of organisms such as bacteria, fungi, viruses, and other genetic material in the gut) so that it can successfully overcome the *C. difficile* infection. *FMT* is widely used in medical centres in the US and Canada to treat recurring *C. difficile* infection, and some medical centres and hospitals in Australia also offer *FMT*.

**Previous Donation and Future Willingness to Donate**

**What does stool donation involve?** After signing up to a register (or filling out an expression of interest), people are typically then asked to complete a health questionnaire. If they pass the health questionnaire, they will usually be required to attend an in-person clinical interview and to go through several rounds of rigorous screening that involves blood and stool tests. People who pass all tests are then cleared to be a stool donor. Stool donors are asked to donate several times because their stool goes to a stool bank to ensure that treatments are available for a patient who may need more than one *FMT*, and because donations from one person can treat many patients. In Australia, stool donors are often asked to donate in a treatment centre or collection facility (e.g., a Therapeutic Goods Administration [TGA] approved toilet in a blood donation centre). In some cases, stool donors may be able to collect their stool at home using materials provided by a treatment centre (e.g., container etc.) and then deliver their donation to the centre.

1. Have you previously donated stool for someone who needed a faecal microbiota transplant?

⭘ Yes

⭘ No

⭘ Unsure

1. How willing would you be to donate stool at a collection facility for a **stool bank** for a person you don't know who is sick with *C. difficile*?

Not at all Extremely

willing willing

0 10 20 30 40 50 60 70 80 90 100

Stool banks often ask donors to donate multiple times over a set period of time.

In terms of frequency and length of time, how often and for how many weeks would you be willing to donate?

Please enter text to describe how often and for how long in the boxes below. For example, if I were willing to give 2 times a day for 1 week then I would write "2 times a day" in the first box and "1 week" in the second box. If I were willing to give twice a week for 12 weeks, then I would write "2 times a week" in the first box and "12 weeks" in the second box.

1. How often would you be willing to donate?

________________________________________________________________

1. For how long would you be willing to donate?

________________________________________________________________

1. Imagine you are accepted as a donor to a stool bank and you are willing to be a donor. How possible would it be for you to donate 5 times each week, over a two-week period?

Not at all Extremely

possible possible

0 10 20 30 40 50 60 70 80 90 100

1. How willing would you be to donate stool **at home and deliver your donation** to a stool bank for a person you don't know who is sick with *C. difficile*?

Not at all Extremely

willing willing

0 10 20 30 40 50 60 70 80 90 100

Stool banks often ask donors to donate multiple times over a set period of time.

In terms of frequency and length of time, how often and for how many weeks would you be willing to donate?

Please enter text to describe how often and for how long in the boxes below. For example, if I were willing to give 2 times a day for 1 week then I would write "2 times a day" in the first box and "1 week" in the second box. If I were willing to give twice a week for 12 weeks, then I would write "2 times a week" in the first box and "12 weeks" in the second box.

1. How often would you be willing to donate?

________________________________________________________________

1. For how long would you be willing to donate?

________________________________________________________________

1. How willing would you be to donate stool to a **loved one** who is sick with *C. difficile*?

Not at all Extremely

willing willing

0 10 20 30 40 50 60 70 80 90 100

1. How willing would you be to donate stool to a **research study** for people who are sick with *C. difficile*?

Not at all Extremely

willing willing

0 10 20 30 40 50 60 70 80 90 100

1. How willing would you be to donate stool to **develop new treatments or a cure** for *C. difficile*?

Not at all Extremely

willing willing

0 10 20 30 40 50 60 70 80 90 100

**Facilitators and Barriers to Donating Stool to a Stool Bank**

1. What would **stop you or make it difficult** to donate stool to a stool bank for a person you don’t know who is sick with *C. difficile*?

________________________________________________________________

________________________________________________________________

________________________________________________________________

________________________________________________________________

________________________________________________________________

**Knowledge Part II**

1. If you were to consider donating stool to a stool bank for a person you don’t know who is sick with *C. difficile*, how important would each of the following be?

|  | Not at all important  1 | 2 | 3 | 4 | 5 | 6 | Very important  7 |
| --- | --- | --- | --- | --- | --- | --- | --- |
| Understanding the aim of donating stool | ⭘ | ⭘ | ⭘ | ⭘ | ⭘ | ⭘ | ⭘ |
| Understanding how donating stool could help you now or in the future | ⭘ | ⭘ | ⭘ | ⭘ | ⭘ | ⭘ | ⭘ |
| Understanding how donating stool could help patients now or in the future | ⭘ | ⭘ | ⭘ | ⭘ | ⭘ | ⭘ | ⭘ |
| Receiving compensation for donating stool | ⭘ | ⭘ | ⭘ | ⭘ | ⭘ | ⭘ | ⭘ |
| The out of pocket cost of donating stool, if any | ⭘ | ⭘ | ⭘ | ⭘ | ⭘ | ⭘ | ⭘ |
| How convenient donating stool would be in terms of logistics | ⭘ | ⭘ | ⭘ | ⭘ | ⭘ | ⭘ | ⭘ |
| Whether you would have to travel in order to donate stool | ⭘ | ⭘ | ⭘ | ⭘ | ⭘ | ⭘ | ⭘ |
| That all information held about you at the stool bank is confidential | ⭘ | ⭘ | ⭘ | ⭘ | ⭘ | ⭘ | ⭘ |
| Understanding every step of the stool donation process before you donate stool | ⭘ | ⭘ | ⭘ | ⭘ | ⭘ | ⭘ | ⭘ |

**Attitude**

1. What is your general attitude towards donating stool to a stool bank?

⭘ Very positive

⭘ Positive, with reservations

⭘ Generally negative but realise it is necessary

⭘ Negative

⭘ No Opinion

1. What is your attitude towards **personally** donating your stool to a stool bank?

⭘ Positive, would generally like to donate

⭘ Neutral, would depend on the situation

⭘ Negative, would generally not want to donate

**Motives**

1. If you were willing to donate stool to a stool bank for a person you don’t know who is sick with *C. difficile*, what would be your **main reasons** for doing so?

________________________________________________________________

________________________________________________________________

________________________________________________________________

________________________________________________________________

________________________________________________________________

**Participant ID**

1. PA_ID Please enter your Prolific ID in the box below. Prolific IDs have 24 alphanumeric characters:

________________________________________________________________

**Thank you for your time in completing this survey.**

If you wish to do so, below is a space for you to provide any additional comments you may have regarding stool donation, faecal microbiota transplantation, or the survey. Otherwise, please proceed to the end of the survey.

________________________________________________________________

________________________________________________________________

________________________________________________________________

________________________________________________________________

________________________________________________________________

⭘ Proceed to the end of the survey

⭘ I would like to withdraw my data
