# Supplementary material for: Determinants of community members’ willingness to donate stool for faecal microbiota transplantation
Source: PLoS One. 2020 Dec 10;15(12):e0243751. doi: 10.1371/journal.pone.0243751 (PMC7728237; doi:10.1371/journal.pone.0243751)
Supplement: S2 Appendix — (DOCX) [file pone.0243751.s002.docx]

**S2 Appendix. Information presented to participants about CDI, FMT, and stool donation.**

**Clostridiodes difficile**

Clostridioides difficile (also referred to as Clostridium difficile, **C. difficile**, or *C. diff*) is a bacteria (germ) that normally lives in your intestines (gut). Sometimes, *C. difficile* grows out of control and can cause infection. Infection can result in frequent diarrhoea for more than a few days, feeling sick to your stomach, stomach pain, and fever. For most people the effects of a *C. difficile* infection are relatively mild, but it can sometimes lead to serious illness and even death. A *C. difficile* infection is normally treated with antibiotics. However, for some people, antibiotics don’t work or the infection returns many times. People who are most at risk for *C. difficile* infection include people who have taken antibiotics, are being treated for cancer, are staying in a hospital, live in a nursing home, have had surgery on their stomach or intestines, or have a weakened immune system.

**Faecal microbiota transplantation**

Faecal microbiota transplantation (**FMT**) involves the transfer of stool from a healthy person to a person with disease in order to treat their disease. It can be a life-saving treatment for people who have recurring *C. difficile* infection. *FMT* has been shown in randomised controlled trials to cure up to 90% of people who have recurring *C. difficile* and for whom antibiotics do not work as a treatment. New research shows that *FMT* may also help to treat people with other conditions such as ulcerative colitis. During the transplant, pre-prepared stool from a carefully screened, healthy person who has donated stool to a stool bank, is transplanted into the colon of the patient. The transplant usually happens by colonoscopy or capsules. *FMT* is thought to work by replacing the patient’s gut microbiome (community of organisms such as bacteria, fungi, viruses, and other genetic material in the gut) so that it can successfully overcome the *C. difficile* infection. *FMT* is widely used in medical centres in the US and Canada to treat recurring *C. difficile* infection, and some medical centres and hospitals in Australia also offer *FMT*.

**What does stool donation involve?**

After signing up to a register (or filling out an expression of interest), people are typically then asked to complete a health questionnaire. If they pass the health questionnaire, they will usually be required to attend an in-person clinical interview and to go through several rounds of rigorous screening that involves blood and stool tests. People who pass all tests are then cleared to be a stool donor. Stool donors are asked to donate several times because their stool goes to a stool bank to ensure that treatments are available for a patient who may need more than one *FMT*, and because donations from one person can treat many patients. In Australia, stool donors are often asked to donate in a treatment centre or collection facility (e.g., a Therapeutic Goods Administration [TGA] approved toilet in a blood donation centre). In some cases, stool donors may be able to collect their stool at home using materials provided by a treatment centre (e.g., container etc.) and then deliver their donation to the centre.
