# Supplementary material for: Determinants of community members’ willingness to donate stool for faecal microbiota transplantation
Source: PLoS One. 2020 Dec 10;15(12):e0243751. doi: 10.1371/journal.pone.0243751 (PMC7728237; doi:10.1371/journal.pone.0243751)
Supplement: Attachment — Submitted filename: Response to Reviewers.docx [file pone.0243751.s003.docx]

**Response to Reviewers – PONE-D-20-21058 Determinants of community members’ willingness to donate stool for faecal microbiota transplantation**

--

Dear Professor Wilson and Reviewers,

Thank you very much for the feedback and for the opportunity to undertake revisions. We appreciate the useful suggestions from the reviewers and have endeavoured to incorporate these where possible. Our responses to reviewer feedback are below. Changes to the manuscript are identified using track changes.

Please note as per our cover letter, since our original PLOS ONE submission we subsequently published an article in Transfusion that used a subset of participants to explore a wholly different focus on the role of ambivalence in decisions about stool donation and used an independent set of variables. We have noted the link to the Transfusion paper in the text of our revised PLOS ONE manuscript (p. 6).

We have separately addressed in our cover letter the additional journal requirements outlined in the manuscript decision letter.

Thank you once again for considering our manuscript and we look forward to hearing from you.

Yours sincerely,

The Authors

--

| **Reviewer 1** |  |
| --- | --- |
| **Feedback** | **Response** |
| *I have very little concerns with the study design, the analysis, the presentation of data and the interpretation – these are all great.* | Thank you very much for your kind evaluation of our work. |
| *There are just a few smaller factors that I think are also worthy of consideration/ amendment:* |  |
| *a) Title – I think should be members’ rather than member’s.* | a) Thank you, the title has now been corrected. |
| *b) Consistency required with spelling, e.g. ‘faecal’ in title but ‘fecal’ in abstract.* | b) Our apologies for the error and thank you for noting. We have now ensured all spelling is consistent. |
| *c) Could you explain a little more about how people were recruited to take the survey? It is important to have this information to give more insight into the population completing it, and any potential biases in participant recruitment.* | c) Thank you for this suggestion. We have added further information in the Method and Materials section on p. 5 and p. 6 about how we recruited participants to do the survey. Specifically, we have noted the approximate number of Australians who were active on the system, the proportion of men, and that participants self-selected into the study and completed a pre-screening survey to confirm their eligibility before being invited to complete the main survey. Pre-screening questions have also been included as Supplementary Information (S1 Appendix). |
| *d) Was any information recorded about work of participants? It would be interesting to see if a connection to healthcare influenced outlook about this.* | d) We agree this information would be interesting to have. Unfortunately, we did not ask participants about their occupation and we have now noted this as a limitation on p. 35. |
| *e) I am not sure that any data is provided regarding where in Australia that participants came from. It might at least be interesting to know if there was any difference in outlook between people in smaller/ more rural communities from larger/urban connurbations? Where differences exist between outcome in this study compared to previous studies from other countries/ regions – could the investigators propose any country-specific regions why these might differ, e.g. aspects related to the delivery of healthcare systems?* | e) Thank you for this suggestion. We agree it would be interesting to explore this aspect. Unfortunately, we did not collect data about location, however we have included this as a limitation based on the potential for differences across region or countries on p. 35. |
| **Reviewer 2** |  |
| **Feedback** | **Response** |
| *This is an interesting and practical survey on potential donors’ attitudes and willingness to FMT.* | Thank you, we appreciate your kind evaluation of our work. |
| 1. *This mainly reflected the situation in Australia. However, the methods and protocol on donor screening in the global varies a lot in different areas or countries. I suggest authors to discuss and the latest consensus report from the FMT-standardization study group. The recommended donor population is younger than the mean age of investigated population in the current study. Importantly, the location for collecting stool is only in the specific room within the FMT center. This is the biosafety requirement according to the consensus. At least this is very important for FMT center. I suggest discuss the direction and different choices under the different regulations. Readers of the journal should know the general view on the donor screening and related laboratory process.* | 1. Thank you for raising this important point. We have now included discussion that screening criteria and protocols vary globally and have included an example of this with references to protocols including the suggested Nanjing consensus from the FMT-standardization Study Group on p. 5-6.   We have also included information that there is variability in stool collection procedures so that readers are aware and noted that this variability in process may impact willingness and acceptability of donation (p. 4 and p. 35) |
| 2. *Line 116 - Information presented to participants about CDI and FMT was particularly important. I would kindly ask the authors to provide the detailed short paragraphs describing CDI and FMT (even as Supplementary material).* | 2. On the Reviewer’s advice, we have now provided the detailed short paragraphs about CDI and FMT as Supplementary Information (S2 Appendix). |
| 3. *Question about Table 1 - The data of BMs daily frequency <1 and BMs weekly frequency <7 are inconsistent, which you explained in the discussion section may be due to inaccurate assessment of participants. This raises questions about the reliability of the questionnaire data.* | 3. Thank you for raising this point. We have now further explained that this inaccuracy in estimating weekly BMs is not unique to our study and has occurred and been noted in prior research (p. 33). It was our knowledge of this research that prompted us to include multiple assessments so readers could obtain a fuller picture about the bowel habits of participants. |
| 4. *Line 178 - Can the author describe the scoring rules for willingness to donate stool in detail?* | 4. We have included further detail about the scoring of willingness on p. 7. |
| 5. *According to the latest report, FMT has been named washed microbiota transplantation (WMT) based on the latest technology. Participants may make different choices after learning about these new developments. You can add these to the discussion section.* | 5. Thank you for this suggestion. We have now noted the following in the Discussion on p. 35:   “Country-specific differences in protocols (e.g., age, facilities) and changes in terminology and processes due to advances in automated facilities (e.g., washed microbiota transplantation vs. manual faecal microbiota transplantation [19]) may also impact willingness and acceptability of stool donation.” |
